# Evolution analysis of low-carbon cooperation of service providers based on Moran process in cloud manufacturing

**DOI:** 10.1371/journal.pone.0299952

**Published:** 2024-03-21

**Authors:** Tiaojuan Han, Jianfeng Lu, Hao Zhang, Wentao Gao

**Affiliations:** CIMS Research Center, Tongji University, Shanghai, China; University of Madeira / NOVA Lincs, PORTUGAL

## Abstract

Low-carbon cooperation among cloud manufacturing service providers is one way to achieve carbon peak and neutrality. Such cooperation is related to the benefits to service providers adopting low-carbon strategies and stochastic factors such as government low-carbon policies, providers’ environmental awareness, and demanders’ low-carbon preferences. Focusing on the evolutionary process of service providers’ low-carbon strategy selection under uncertain factors, a stochastic evolutionary game model is constructed based on the Moran process, and the equilibrium conditions for low-carbon cooperation among providers are analyzed under benefit-dominated and stochastic factor-dominated situations. Through numerical simulation, the effects of the cloud platform’s cost-sharing coefficient for low-carbon investment, matching growth rate, carbon trading price, and group size on providers’ low-carbon strategy evolution are analyzed. The research results show that increasing the cloud platform’s low-carbon cost-sharing, carbon trading price, and group size can promote low-carbon cooperation among service providers. With greater low-carbon investment costs and greater stochastic factor interference, the providers’ enthusiasm for low-carbon cooperation decreases. This study fills the research gap in the low-carbon cooperation evolution of cloud manufacturing providers based on the stochastic evolutionary game and provides decision-making suggestions for governments and cloud platforms to encourage provider participation in low-carbon cooperation and for providers to adopt low-carbon strategies.

## 1 Introduction

Cloud manufacturing is an advanced manufacturing mode proposed by Li Bohu in 2010 [1]. It integrates dispersed manufacturing resources and capabilities for centralized management through advanced technologies [2] and shares manufacturing services on demand, including processing services and logistics services. Three main stakeholders participate in cloud manufacturing: service providers, service demanders, and cloud platforms. Specifically, service providers publish idle manufacturing resources and capabilities on the cloud platform through virtualization, service demanders publish service demands on the cloud platform [3], and the cloud platform matches services and demands.

Cloud manufacturing provides a resource-sharing platform and new ways for service providers to cooperate in reducing carbon emissions and achieving carbon peak and neutrality. Service providers actively participate in low-carbon cooperation through technological R&D, equipment upgrading, etc., which produces economic value and improves market competitiveness. For example, Siemens provided low-carbon emission services to address the high energy consumption and carbon emissions of Longmen Iron and Steel Plant, and the provided frequency conversion transformation technology of the sintering machine brought more than 9 million yuan in energy-saving and emission reduction every year [4]. However, service providers engaging in low-carbon cooperation must pay low-carbon costs, such as introducing low-carbon technologies and purchasing low-carbon equipment. Furthermore, the low-carbon investment process might offer other providers free rides, rendering most service providers reluctant to provide low-carbon manufacturing services based on their own interests. In addition, the low-carbon cooperation of service providers is affected by various stochastic factors, such as COVID-19, the Sino-U.S. trade war [5], and providers’ environmental awareness, which brings uncertainties. Providers weigh benefits, costs, risks, and other factors before adopting low-carbon strategies. Therefore, providers’ low-carbon cooperation is a long-term dynamic game process with gains and losses. Providers cannot choose the optimal strategy at the beginning and finally achieve it through continuous observation, learning, and imitation. To promote low-carbon cooperation among service providers, the low-carbon incentives of the cloud platform are a key factor for low-carbon cooperation among service providers. Therefore, it is necessary to analyze providers’ low-carbon strategy selection under the interference of stochastic factors and discuss the impacts of parameters such as the platform’s incentives on the providers’ low-carbon strategy evolution.

In low-carbon supply chain operation management, carbon emissions are a constraint in low-carbon supply chain design and planning [6]. Scholars have analyzed the carbon emission reduction coordination and production/pricing decisions based on classical game theory [7, 8], and previous studies analyzed stakeholder low-carbon behavior under carbon emission reduction policies based on the evolutionary game theory [9, 10]. In terms of research content, the above research analyzed the carbon emission reduction strategies of enterprises but not the low-carbon cooperation among cloud manufacturing service providers. In terms of research methods, classical game theory-based research on low-carbon decision-making in supply chain operation management did not consider the dynamic decision-making process of the stakeholders. Evolutionary game theory does not apply to analyzing the impact of stochastic factors on the low-carbon strategy evolution of a limited number of providers.

In summary, service providers have different and limited information acquisition and processing capabilities when making decisions, and their decision-making behavior is not completely rational due to the influence of stochastic factors. Service providers adjust their strategies through information interaction and imitation learning, and a strategy is replaced by a dominant one. This process can be expressed with a Moran model. The Moran process introduces Darwin’s evolutionary theory into the evolutionary game and, unlike the evolutionary game, considers the selection intensity, which is divided into strong selection and weak selection according to the dependence between individual payoff and fitness [11]. This study analyzes the low-carbon strategy evolution of service providers under different selection intensities, reflecting the randomness and dynamics in the low-carbon decision-making process.

Taking the service provider group as the research object, this study analyzes the evolutionary process of providers’ low-carbon strategy selection considering stochastic factors. Under government carbon trading policies, a stochastic evolution game model is constructed through the Moran process, in which stochastic factors and expected benefits dominate. The effects of system factors such as low-carbon investment cost, group size, and selection intensity and low-carbon intervention factors such as low-carbon cost-sharing, service matching growth rate, and carbon trading price on providers’ low-carbon cooperation rate are analyzed. This study mainly solves the following two problems: What are the equilibrium conditions for providers’ low-carbon cooperation under expected benefits-dominated and stochastic factor-dominated situations? How do providers’ low-carbon strategies evolve under the effects of different factors?

This research has the following contributions: (1) Compared with the static model of low-carbon cooperation in the supply chain, this study considers the randomness and dynamics of the low-carbon cooperation process and describes the low-carbon strategy evolution under the influence of stochastic factors, thus filling the research gap in low-carbon cooperation among cloud manufacturing service providers. (2) Compared with evolutionary games based on replicator dynamic equations, the Moran process considers the limited size of provider groups and the interference degree of stochastic factors (selection intensity). Based on the Moran process, a stochastic evolutionary game model is constructed for the low-carbon cooperation among provider groups.

The remainder of this article is structured as follows. Section 2 reviews the relevant literature. Section 3 establishes the stochastic evolutionary game model of low-carbon cooperation of cloud manufacturing service providers. Section 4 explores the conditions for adopting low-carbon strategies under strong selection and weak selection. The impacts of different factors on the low-carbon strategy evolution of service providers are analyzed in Section 5. Section 6 presents the discussions. In Section 7, conclusions and suggestions are proposed.

## 2 Related work

This research investigates the carbon emission reduction coordination mechanism and production/pricing decision-making and explores the carbon emission reduction behaviors of supply chain members under carbon emission reduction policies.

### 2.1 Carbon emission reduction coordination mechanism and production/pricing decision-making

Supply chain members adopting low-carbon strategies may not be able to reduce emissions independently due to huge low-carbon costs. Therefore, cooperative emission reduction among supply chain members is an effective way to reduce carbon emissions [12]. Supply chain members jointly develop low-carbon green innovative technologies, share low-carbon investment costs, and benefit from carbon emission reduction.

Researchers have studied the coordination relationship of low-carbon cooperation among supply chain members from the perspectives of revenue sharing and cost-sharing. Wu et al. [8] considered manufacturer financing strategies and designed a cost-sharing contract between the manufacturer and the retailer through the game theory for their coordination. Yang et al. [13] built a joint carbon emission reduction decision-making model for cloud platform operators and service providers based on the Stackelberg game theory and introduced a side-payment contract to coordinate supply chain members in environmental governance.

In terms of carbon emission reduction production/pricing decisions, Chen et al. [14] analyzed the pricing and emission reduction decisions of two competing manufacturers through the non-cooperative game theory. Ji et al. [15] analyzed the decision-making of carbon emission reduction rates and low-carbon promotion levels under different power models of manufacturers and retailers based on the Stackelberg game theory. Scholars have analyzed the carbon emission reduction and pricing decisions under government carbon policies. Cai et al. [6] analyzed the pricing and carbon emission reduction decisions under carbon trading supervision based on the differential game model. Considering carbon tax policies, Zhang et al. [16] considered manufacturer competition and cooperation and explored manufacturer operating strategies for low-carbon products.

However, the above research assumes that stakeholders are completely rational and establishes a static game optimization model to analyze the short-term optimal decision-making of supply chain enterprises [17]. The low-carbon decision-making of supply chain members is a long-term dynamic process [18]. The environment is more complex and uncertain, and enterprises have information asymmetry and limited rationality. As a result, enterprises can not maximize their own profits in a game but constantly observe and update strategies through exchanging information and imitating other players. In short, classical game theory cannot describe the dynamic stakeholder decision-making process.

### 2.2 Carbon emission reduction behaviors of supply chain members under carbon emission reduction policies

Researchers also analyzed the low-carbon strategy selection behavior among governments, enterprises, and consumers based on the evolutionary game [9]. Chen et al. [19] considered green and low-carbon technological innovation and constructed a tripartite evolutionary game model among the government, the public, and enterprises. The effects of government carbon emission reduction policies on the decision-making of supply chain stakeholders were also studied [20]. By modeling the tripartite evolutionary game for the low-carbon technology innovation among enterprises, governments and customers, Yuan et al. [21] analyzed the impact of regulatory intensity and government subsidies on strategy evolution. Considering the different government incentives and regulations, Chen et al. [22] studied the green R&D diffusion in the photovoltaic industry.

Although regarded as a game participant, the government in most previous studies cannot master the specific production information of enterprises or effectively and timely supervise and restrict violations [23]. Compared with traditional transactions, cloud platforms can motivate service providers and demanders to participate in low-carbon cooperation. However, few studies have considered the impact of the cloud platform on enterprises’ emission reduction behaviors. In addition, previous literature analyzed enterprises’ low-carbon and emission reduction strategies based on the evolutionary game theory but neglected the impacts of uncertain factors on the strategy equilibrium. In practice, the number of service providers is finite, and their strategy selection is affected by various stochastic factors, such as the environmental awareness of providers, which conforms to the stochastic evolutionary game theory. It is of practical significance to regard the evolution process of service providers’ low-carbon decision-making as a stochastic process [24].

## 3 Stochastic game model

### 3.1 Research framework

Service providers in the cloud manufacturing system are homogeneous, and the group size is finite (let the group size be *N*). Service providers obtain limited information about low-carbon investment costs and market demands and constantly adjust their strategies through information interaction and game learning. To simplify the analysis, the service provider group size is assumed to remain unchanged [25]. The strategy set for service providers is {providing low-carbon manufacturing services(LS), providing traditional manufacturing services(TS)}. LS means that providers provide manufacturing services that reduce carbon emissions, such as optimizing the machining process. TS means that providers provide manufacturing services with the original process and technology. Individuals change their strategies by interacting among themselves. When all service providers adopt LS, LS spreads to the entire group, i.e., LS takes root, and low-carbon cooperation succeeds. Otherwise, TS takes root, and low-carbon cooperation fails.

**Fig 1:**
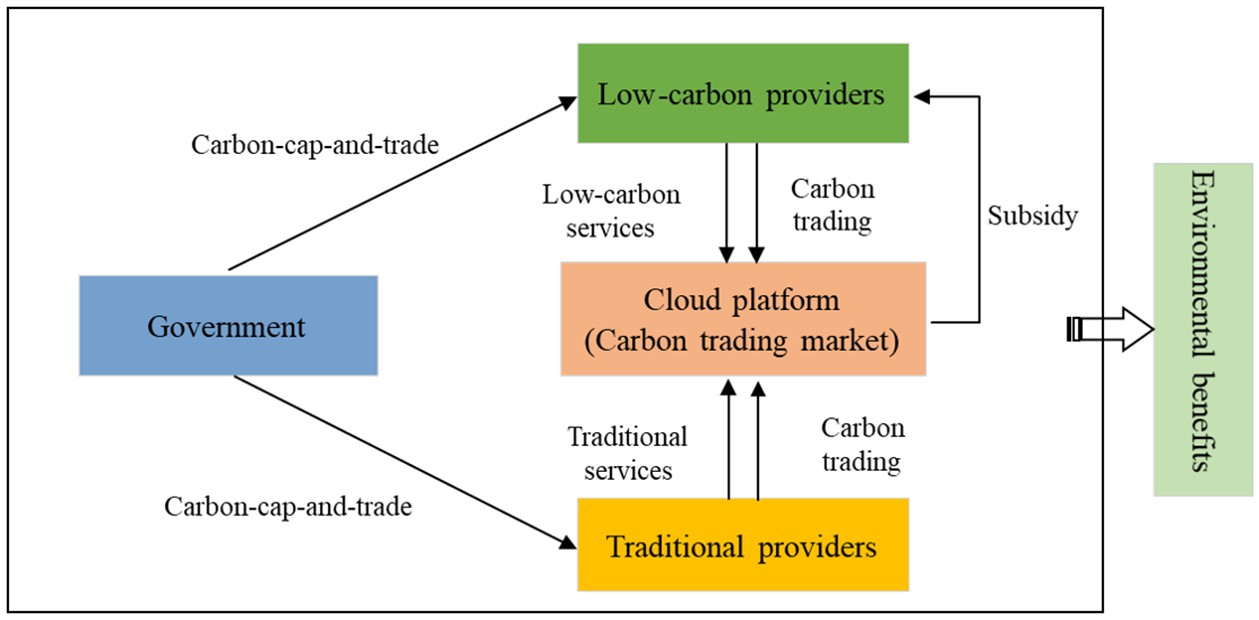
Research framework.

Currently, carbon cap-and-trade mechanisms are commonly implemented, where the government sets carbon emissions caps for enterprises. Enterprises can sell surplus carbon emission permits on carbon trading markets when not reaching their carbon quota [26]. Service providers providing low-carbon services often have carbon emissions below their carbon quota, allowing them to benefit from surplus carbon emission permit sales. In addition, such providers greatly improve the reputation of the cloud platform, thus improving platform benefits by attracting service demanders with environmental awareness. To encourage low-carbon investment by service providers, the cloud platform shares the low-carbon investment costs of service providers. This study analyzes whether cloud manufacturing service providers provide low-carbon manufacturing services under government carbon trading policies. The research framework is shown in Fig 1.

### 3.2 Theoretical basis

Assume that *N* individuals in a group with the strategy set {A, B} engage in a pairwise game, the payoff matrix of the game is shown in Table 1 [27].

**Table 1:** Payment matrix.

	A	B
A	(*a*,*a*)	(*b*,*c*)
B	(*c*,*b*)	(*d*,*d*)

Let the number of individuals choosing strategy A be *i*, the benefits of choosing strategy A are expressed in Eq (1), and the benefits of choosing strategy B are calculated in Eq (2).

$$\begin{array}{c} \pi_{i}^{A}=\frac{a(i-1)+b(N-i)}{N-1},i=1,2,\cdots ,N \end{array}$$ (1)

$$\begin{array}{c} \pi_{i}^{B}=\frac{ci+d(N-i-1)}{N-1},i=0,1,\cdots ,N-1 \end{array}$$ (2)

The Moran process includes strong selection and weak selection according to the relationship between individual payoff and individual fitness. The mapping relationship between fitness and payoff is an exponential or linear function. The linear mapping is suitable for weak selection and has limitations in strong selection analysis [28]. Essentially the same as linear mapping, exponential mapping is suitable for strong selection and weak selection [29, 30]. Therefore, the fitness is assumed to be an exponential function of the payoff, as expressed in Eqs (3) and (4).

$$\begin{array}{c} f_{i}^{A}=e^{w\pi_{i}^{A}} \end{array}$$ (3)

$$\begin{array}{c} f_{i}^{B}=e^{w\pi_{i}^{B}} \end{array}$$ (4)

where *w* denotes selection intensity and illustrates the contribution of game payoff to fitness, 0 ≤ *w* ≤ 1. *w* = 0 indicates a neutral selection, and the expected payoff has no effect on the fitness. *w* << 1 indicates weak selection, where the expected payoff has a very small effect on fitness, and the fitness depends almost entirely on stochastic factors [31]. *w* = 1 indicates a strong selection with the fitness completely determined by the expected payoff.

**Fig 2:**
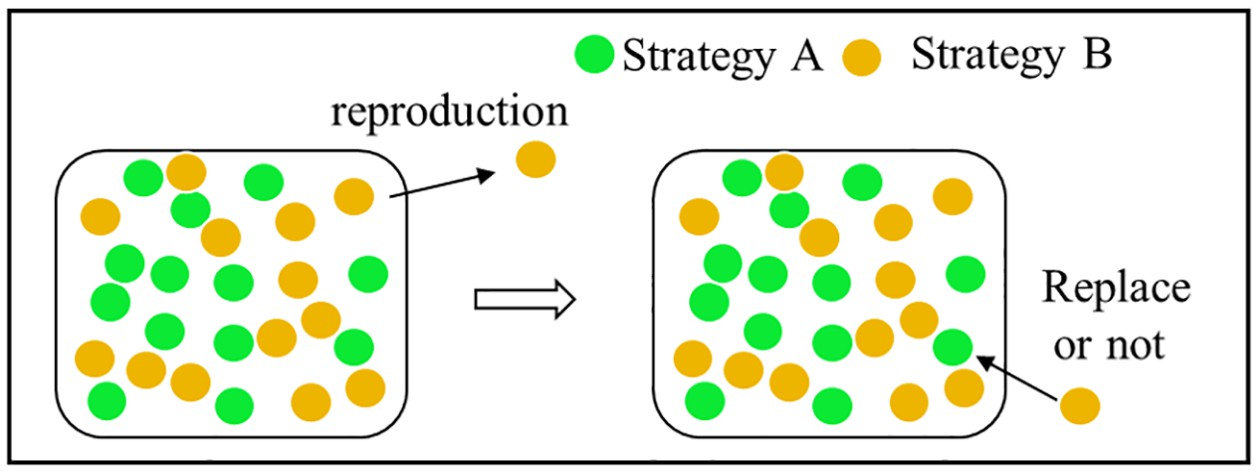
Strategy update process based on Moran process.

The strategy update process based on the Moran process is divided into three steps: compare, reproduce, and replace [11, 32], as shown in Fig 2. At each time step, an individual is selected to produce an offspring according to the individual’s fitness. The offspring replaces another individual stochasticly selected in a group, and the total group size remains unchanged [17].

**Fig 3:**
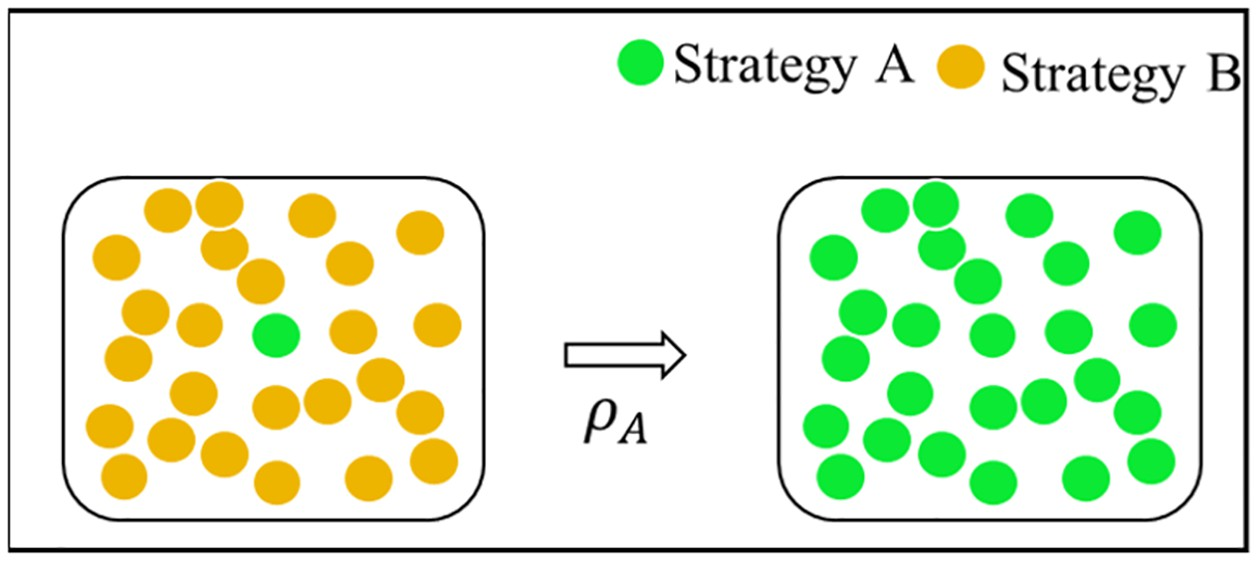
Fixed probability of strategy A.

The strategy selection under natural selection is characterized by fixed probabilities. The fixed probability of strategy A is defined as follows: One individual chooses strategy A in the initial group. The probability that all individuals choose strategy A after evolution is expressed as *p*_*A*_, as shown in Fig 3. Similarly, the fixed probability *p*_*B*_ of strategy B can be obtained.

If *a* = *b* = *c* = *d* in Table 1, *p*_*A*_ = *p*_*B*_ = 1/*N*, and 1/*N* is the probability under neutral selection, we have the following theorem.

**Theorem 1.** If $p_{A}>\frac{1}{N}$, natural selection favors strategy A over strategy B.

if $p_{A}<\frac{1}{N}$, natural selection does not favor strategy A over strategy B.

if $p_{B}>\frac{1}{N}$, natural selection favors strategy B over strategy A.

if $p_{B}<\frac{1}{N}$, natural selection discourages replacing strategy A with strategy B.

### 3.3 Model assumptions

The main parameters for the stochastic evolution model are shown in Table 2. The basic assumptions and the game process of service providers can be divided into the following situations.

1. With a strategy combination of {TS,TS}, *N* service providers provide traditional manufacturing services. The basic benefit to service providers providing manufacturing services is *G*_1_[33]. The carbon quota for service providers is *μ*_*p*_. When all providers adopt TS, their carbon emission is *μ*_*h*_, *μ*_*h*_ > *μ*_*p*_. As no service providers adopt low-carbon technologies, extra carbon emissions permits are purchased at the price *P*_*c*_ from the carbon trading market [34, 35]. The same assumption can be found in [33–35]. Therefore, when all service providers adopt TS, service providers’ payment is (*G*_1_ − *P*_*c*_(*μ*_*h*_ − *μ*_*p*_), *G*_1_ − *P*_*c*_(*μ*_*h*_ − *μ*_*p*_)).
2. With a strategy combination of {LS, LS}, *N* service providers provide low-carbon manufacturing services. The low-carbon cost of service providers choosing LS is *C*_1_, which includes expenditures for improving production processes and purchasing environmental protection equipment [36]. Low-carbon manufacturing services bring additional benefits to the cloud platform, such as improved reputation, which attracts more service providers and demanders. To encourage service providers to adopt LS, the cloud platform offers low-carbon cost subsidy *αC*_1_ to service providers. When providers adopt LS, their carbon emission is *μ*_*l*_, *μ*_*l*_ < *μ*_*p*_. Service providers sell surplus carbon quota to other providers at the price *P*_*c*_, improving their profits [34, 35]. Similar assumptions can be found in [33–36]. When all service providers adopt low-carbon manufacturing services, the payment is *G*_1_ − *C*_1_ + *αC*_1_ + *P*_*c*_(*μ*_*p*_ − *μ*_*l*_), *G*_1_ − *C*_1_ + *αC*_1_ + *P*_*c*_(*μ*_*p*_ − *μ*_*l*_).
3. With a strategy combination of {LS, TS}, the matching rate is introduced in the game process of service providers according to [36]. When providers provide low-carbon manufacturing services, the growth rate of demand matching is *δ*. When providers choose TS, the reduction rate of providers’ demand matching is *δ*. The matching rate affects providers’ benefits and carbon emissions. (1) The extra benefit of providers choosing LS is *δG*_1_, and rising carbon emission is *δμ*_*l*_. Therefore, the basic benefit of providers choosing LS is (1 + *δ*)*G*_1_, and the benefit from selling surplus carbon quota *μ*_*p*_ − (1 + *δ*)*μ*_*l*_ at the price *P*_*c*_ is *P*_*c*_[*μ*_*p*_ − (1 + *δ*)*μ*_*l*_]. In addition, providers providing low-carbon manufacturing services obtain low-carbon cost subsidies *αC*_1_ from the cloud platform and bear low-carbon investment cost *C*_1_. (2) When providers choose TS, their reduced benefit is *δG*_1_, and their reduced carbon emission is *δμ*_*h*_. Therefore, the basic benefit of providers choosing TS is (1 − *δ*)*G*_1_, and the cost for carbon emission permits (1 − *δ*)*μ*_*h*_ − *μ*_*p*_ at the price *P*_*c*_ is *P*_*c*_[(1 − *δ*)*μ*_*h*_ − *μ*_*p*_]. Based on the above analysis, the payment of service providers is (1 + *δ*)*G*_1_ − *C*_1_ + *αC*_1_ + *P*_*c*_[*μ*_*p*_ − (1 + *δ*)*μ*_*l*_], (1 − *δ*)*G*_1_ − *P*_*c*_[(1 − *δ*)*μ*_*h*_ − *μ*_*p*_].

**Table 2:** Main parameters and meanings.

Symbols	Meanings
*G*_1_	Basic benefit of service providers providing low-carbon manufacturing services
*C*_1_	Low-carbon investment cost of service providers choosing LS
*δ*	Growth rate of demand matching when providers choose LS, 0 ≤ *δ* ≤ 1
*α*	Sharing coefficient of low-carbon cost, 0 ≤ *α* ≤ 1
*μ*_*p*_	Carbon quota for service providers
*μ*_*l*_	Carbon emission of service providers choosing LS
*μ*_*h*_	Carbon emission of service providers choosing TS
*P*_*c*_	Carbon trading price of unit carbon emission

The payment matrix of service providers choosing different strategies is listed in Table 3.

**Table 3:** Payment matrix of service providers.

Providers	LS	TS
LS	*G*_1_ − *C*_1_ + *αC*_1_ + *P*_*c*_(*μ*_*p*_ − *μ*_*l*_) *G*_1_ − *C*_1_ + *αC*_1_ + *P*_*c*_(*μ*_*p*_ − *μ*_*l*_)	(1 + *δ*)*G*_1_ − *C*_1_ + *αC*_1_ + *P*_*c*_[*μ*_*p*_ − (1 + *δ*)*μ*_*l*_] (1 − *δ*)*G*_1_ − *P*_*c*_[(1 − *δ*)*μ*_*h*_ − *μ*_*p*_]
TS	(1 − *δ*)*G*_1_ − *P*_*c*_[(1 − *δ*)*μ*_*h*_ − *μ*_*p*_] (1 + *δ*)*G*_1_ − *C*_1_ + *αC*_1_ + *P*_*c*_[*μ*_*p*_ − (1 + *δ*)*μ*_*l*_]	*G*_1_ − *P*_*c*_(*μ*_*h*_ − *μ*_*p*_) *G*_1_−*P*_*c*_(*μ*_*h*_−*μ*_*p*_)

### 3.4 Model building

With *N* service providers in the system, *i* adopts LS, and *N*−*i* adopts TS. According to the payment matrix in Table 3, the expected payoff of providers providing low-carbon manufacturing services is calculated in Eq (5).

$$\begin{array}{c} \pi_{i}^{L}=\frac{i-1}{N-1}[G_{1}-C_{1}+\alpha C_{1}+P_{c}(\mu_{p}-\mu_{l})]+\frac{N-i}{N-1}\\ \{\left(1+\delta \right)G_{1}-C_{1}+\alpha C_{1}+P_{c}[\mu_{p}-\left(1+\delta \right)\mu_{l}]\},i=1,2,\cdots N \end{array}$$ (5)

The expected payoff of service providers providing traditional manufacturing services is expressed in Eq (6).

$$\begin{array}{c} \pi_{i}^{T}=\frac{i}{N-1}\{\left(1-\delta \right)G_{1}-P_{c}[\left(1-\delta \right)\mu_{h}-\mu_{p}]\}+\frac{N-i-1}{N-1}[G_{1}-P_{c}(\mu_{h}-\mu_{p})]\\ i=0,1,2,\cdots N-1 \end{array}$$ (6)

In addition to the expected payoff (the main factor), some stochastic factors also affect service providers’ low-carbon decision-making. The effect of stochastic factors is simplified as an exogenous variable-natural selection intensity *w* [31].

The fitness of service providers choosing LS is expressed in Eq (7).

$$\begin{array}{c} f_{i}^{L}=e^{w\pi_{i}^{L}} \end{array}$$ (7)

The fitness of service providers choosing TS is calculated in Eq (8).

$$\begin{array}{c} f_{i}^{T}=e^{w\pi_{i}^{T}} \end{array}$$ (8)

where *w* → 0, with weak selection. Various stochastic factors affect service providers’ low-carbon strategy selection, such as government low-carbon policy, providers’ environmental awareness and herd mentality, and demanders’ low-carbon preference. That is, stochastic factors are dominant. When *w* = 1, providers are completely rational. Providers’ fitness is completely determined by the expected benefits of choosing LS/TS and is not affected by other factors. That is, the expected benefits are dominant.

At each time step of the Moran process, the providers’ quantity may increase by one, decrease by one, or remain unchanged [31]. The probability that the service providers choosing LS increase by one is calculated in Eq (9).

$$\begin{array}{c} p_{i,i+1}=\frac{if_{i}^{L}}{if_{i}^{L}+\left(N-i\right)f_{i}^{T}}\frac{N-i}{N} \end{array}$$ (9)

Similarly, the probability that the providers choosing LS decrease by one is expressed in Eq (10).

$$\begin{array}{c} p_{i,i-1}=\frac{\left(N-i\right)f_{i}^{T}}{if_{i}^{L}+\left(N-i\right)f_{i}^{T}}\frac{i}{N} \end{array}$$ (10)

The probability that the service providers choosing LS remain unchanged is derived in Eq (11).

$$\begin{array}{c} p_{i,i}=1-p_{i,i+1}-p_{i,i-1} \end{array}$$ (11)

The changes in providers choosing LS within each time step can be expressed as *i* + 1, *i* − 1, and *i*, and the transition probability from state *i* to other states is 0. Therefore, the transition probability matrix of the Moran process is a tridiagonal one shown in Eq (12). The elements of the principal diagonal and secondary diagonal can be derived with Eqs (9)–(11).

$$\begin{array}{c} p=[\begin{array}{cccccccc} p_{0,0} & p_{0,1} & 0 & \cdots & 0 & 0 & 0 & \\ p_{1,0} & p_{1,1} & p_{1,2} & \cdots & 0 & 0 & 0 & \\ 0 & p_{2,1} & p_{2,2} & p_{2,3} & \cdots & 0 & 0 & \\ \vdots & \vdots & p_{i,i-1} & p_{i,i} & p_{i,i+1} & \vdots & 0 & \\ 0 & 0 & 0 & \cdots & p_{N-1,N-2} & p_{N-1,N-1} & p_{N-1,N}\\ 0 & 0 & 0 & \cdots & 0 & p_{N,N-1} & p_{N,N} \end{array}] \end{array}$$ (12)

The ratio of *p*_*i*,*i*+1_ and *p*_*i*,*i*−1_ is calculated in Eq (13).

$$\begin{array}{c} \delta_{i}=\frac{p_{i,i+1}}{p_{i,i-1}}=\frac{f_{i}^{L}}{f_{i}^{T}}=e^{w(\pi_{i}^{L}-\pi_{i}^{T})} \end{array}$$ (13)

When *δ*_*i*_ → 0, the service providers choosing TS may increase. When *δ*_*i*_ → ∞, the service providers choosing LS may increase. When *δ*_*i*_ → 1, the service providers choosing LS may remain unchanged.

The Moran process includes two absorption states: *i* = 0 and *i* = *N*, indicating all providers choose TS or LS, respectively. After the population reaches the absorption state, the number of providers remains unchanged, and the system no longer evolves as long as the external environment remains unchanged. Low-carbon cooperation of service providers is successful when all providers choose LS.

According to the total probability formula, *i* providers choose LS in the beginning, and the system eventually evolves to a stable state with all providers choosing LS. In this situation, the fixed probability can be expressed in Eq (14).

$$\begin{array}{c} \left\{ \begin{array}{c} x_{0}=1\\ x_{i}=x_{i+1}p_{i,i+1}+x_{i}p_{i,i}+x_{i-1}p_{i,i-1},i=1,\cdots ,N-1\\ x_{N}=1 \end{array}\right. \end{array}$$ (14)

By substituting Eqs (9)–(11) into Eq (14), the fixed probability that *i* providers initially choose LS in the group can be calculated with Eq (15).

$$\begin{array}{c} x_{i}=\frac{1+\sum_{j=1}^{i-1}\;\prod_{k=1}^{j}\;\frac{f_{k}^{T}}{f_{k}^{L}}}{1+\sum_{j=1}^{N-1}\;\prod_{k=1}^{j}\;\frac{f_{k}^{T}}{f_{k}^{L}}} \end{array}$$ (15)

The probability of one provider choosing LS in the initial group and all providers finally choosing LS is a fixation probability of LS denoted as *p*_*L*_.

$$\begin{array}{c} p_{L}=x_{1}=\frac{1}{1+\sum_{j=1}^{N-1}\;\prod_{k=1}^{j}\;\frac{f_{k}^{T}}{f_{k}^{L}}}=\frac{1}{1+\sum_{j=1}^{N-1}\;e^{w\sum_{k=1}^{j}(\pi_{k}^{T}-\pi_{k}^{L})}} \end{array}$$ (16)

The probability of one provider initially choosing TS and all providers finally choosing TS is a fixation probability of TS denoted as *p*_*T*_.

$$\begin{array}{c} p_{T}=1-x_{N-1}=\frac{\prod_{j=1}^{N-1}\;\frac{f_{j}^{T}}{f_{j}^{L}}}{1+\sum_{j=1}^{N-1}\;\prod_{k=1}^{j}\;\frac{f_{k}^{T}}{f_{k}^{L}}}=\frac{1}{1+\sum_{j=1}^{N-1}\;e^{w\Sigma_{k=1}^{j}(\pi_{k}^{L}-\pi_{k}^{T})}} \end{array}$$ (17)

The ratio of *p*_*L*_ and *p*_*T*_ is derived in Eq (18).

$$\begin{array}{c} \gamma =\frac{p_{L}}{p_{T}}=\prod_{j=1}^{N-1}\;\frac{f_{j}^{L}}{f_{j}^{T}}=e^{w\sum_{i=1}^{N-1}(\pi_{i}^{L}-\pi_{i}^{T})} \end{array}$$ (18)

When *γ* > 1, *p*_*L*_ > *p*_*T*_, the number of service providers choosing LS can increase, and LS is more likely to become a stable strategy. By Substituting Eqs (5) and (6) into Eq (18), Eq (19) is obtained.

$$\begin{array}{c} 2(C_{1}-\alpha C_{1}+P_{c}\mu_{l}-P_{c}\mu_{h})+\\ N[2\delta G_{1}-2C_{1}+2\alpha C_{1}+P_{c}(-2\mu_{l}+2\mu_{h}-\delta \mu_{h}-\delta \mu_{l})]>0 \end{array}$$ (19)

Similarly, when *γ* < 1, *p*_*L*_ < *p*_*T*_, the number of service providers choosing TS can increase, and TS is more likely to become a stable strategy.

The pseudo-code of the Moran process is presented in Algorithm 1.

**Algorithm 1** Algorithm of Moran process evolution

**Input:** Group size, payment matrix, selection intensity

**Output:** Group’s current state

1: Initialization: Group state

2: **for** *i* = 0; *i* < *N* + 1; *i* + + **do**

3:  Calculate $\pi_{i}^{L}$ and $\pi_{i}^{T}$ with Eqs (5) and (6)

4:  Calculate $f_{i}^{L}$ and $f_{i}^{T}$ with Eqs (7) and (8)

5:  **if** *i* == 0 or *i* == *N* **then**

6:   *p*_*i*,*i*_ = 1

7:  **else**

8:   Calculate *p*_*i*,*i*+1_, *p*_*i*,*i*−1_, and *p*_*i*,*i*_ based on Eqs (9)–(11)

9: **for** time_steps = 0; time_steps < time_iter; time_steps ++ **do**

10:  Update group’s current state based on the Markov process

## 4 Strategy evolution analysis of service providers under different selection intensities

This section analyzes the low-carbon strategy evolution of service providers under different selection intensities to reflect the randomness and dynamics of low-carbon decision-making.

### 4.1 Strong selection

*w* = 1 indicates strong selection. The fitness of providers is only affected by the expected payoff, and stochastic factors have no effect on the fitness. By comparing the difference *h*_*i*_ in the two strategies’ fitness in each state, the change in the number of service providers adopting LS is analyzed [37].

$$\begin{array}{c} h_{i}=f_{i}^{L}-f_{i}^{T}.i=1,2,\cdots ,N-1 \end{array}$$ (20)

If *h*_1_ > 0, the provider’s selection behavior supports LS to invade TS. If *h*_*N*−1_ < 0, the provider’s selection behavior supports TS to invade LS. When *i* = 1 and *i* = *N* − 1, substituting Eqs (5)–(8) into Eq (20) can yield Eqs (21) and (22).

$$\begin{array}{c} h_{1}=f_{1}^{L}-f_{1}^{T}\\ =e^{w\{\left(1+\delta \right)G_{1}-C_{1}+\alpha C_{1}+P_{c}[\mu_{p}-\left(1+\delta \right)\mu_{l}]\}}-\\ e^{w\frac{1}{N-1}\{\left(1-\delta \right)G_{1}-P_{c}[\left(1-\delta \right)\mu_{h}-\mu_{p}]\}+w\frac{N-2}{N-1}[G_{1}-P_{c}(\mu_{h}-\mu_{p})]}\\ =e^{w\{\left(1+\delta \right)G_{1}-C_{1}+\alpha C_{1}+P_{c}[\mu_{p}-\left(1+\delta \right)\mu_{l}]\}}-e^{w\{[G_{1}-P_{c}(\mu_{h}-\mu_{p})]-\frac{1}{N-1}(\delta G_{1}-\delta P_{c}\mu_{h})\}} \end{array}$$ (21)

$$\begin{array}{c} h_{N-1}=f_{N-1}^{L}-f_{N-1}^{T}\\ =e^{w\frac{N-2}{N-1}[G_{1}-C_{1}+\alpha C_{1}+P_{c}(\mu_{p}-\mu_{l})]+w\frac{1}{N-1}\{\left(1+\delta \right)G_{1}-C_{1}+\alpha C_{1}+P_{c}[\mu_{p}-\left(1+\delta \right)\mu_{l}]\}}\\ -e^{w\{\left(1-\delta \right)G_{1}-P_{c}[\left(1-\delta \right)\mu_{h}-\mu_{p}]\}}\\ =e^{w[G_{1}-C_{1}+\alpha C_{1}+P_{c}(\mu_{p}-\mu_{l})]+w\frac{1}{N-1}(\delta G_{1}-P_{c}\delta \mu_{l})}-e^{w\{\left(1-\delta \right)G_{1}-P_{c}[\left(1-\delta \right)\mu_{h}-\mu_{p}]\}} \end{array}$$ (22)

The game system may have the following scenarios [38].

1. If *h*_1_ > 0, *h*_*N*−1_ > 0, the providers’ selection behavior supports LS to invade TS and is against TS to invade LS. LS becomes an evolutionarily stable strategy.
2. If *h*_1_ > 0, *h*_*N*−1_ < 0, the number of providers adopting LS is growing, and the providers’ selection behavior supports LS to invade TS. The number of *N* − 1 providers adopting LS is decreasing, and the behavior supports TS to invade LS. The hybrid strategy is an evolutionarily stable strategy, and providers’ selection behavior tends to change.
3. If *h*_1_ < 0, *h*_*N*−1_ > 0, the selection behavior is against mutual invasion between LS and TS. The evolutionarily stable strategy is a hybrid strategy, and the selection behavior is against change.
4. If *h*_1_ < 0, *h*_*N*−1_ < 0, the number of providers choosing LS is decreasing, and the providers’ selection behavior is against LS to invade TS. Similarly, the number of *N* − 1 providers choosing LS is decreasing, and the selection behavior favors TS to invade LS. TS is an evolutionarily stable strategy.

Based on Eqs (21) and (22), two propositions can be obtained.

**Proposition 1.** When *δG*_1_ − *C*_1_ + *αC*_1_ + *P*_*c*_[*μ*_*h*_ − (1 + *δ*)*μ*_*l*_] > 0, *δG*_1_ − *C*_1_ + *αC*_1_ + *P*_*c*_[(1 − *δ*)*μ*_*h*_ − *μ*_*l*_] > 0, we have *N*_1_ that $N_{1}=\text{max}\;(\frac{-C_{1}+\alpha C_{1}+P_{c}[\left(1+\delta \right)\mu_{h}-\left(1+\delta \right)\mu_{l}]}{\delta G_{1}-C_{1}+\alpha C_{1}+P_{c}[\mu_{h}-\left(1+\delta \right)\mu_{l}]},\frac{-C_{1}+\alpha C_{1}+P_{c}[\left(1-\delta \right)\mu_{h}-\left(1+\delta \right)\mu_{l}]}{\delta G_{1}-C_{1}+\alpha C_{1}+P_{c}[\left(1-\delta \right)\mu_{h}-\mu_{l}]})$. When *N* > *N*_1_, LS becomes an evolutionarily stable strategy. There is *N*_2_, $N_{2}=\text{min}\;(\frac{-C_{1}+\alpha C_{1}+P_{c}[\left(1+\delta \right)\mu_{h}-\left(1+\delta \right)\mu_{l}]}{\delta G_{1}-C_{1}+\alpha C_{1}+P_{c}[\mu_{h}-\left(1+\delta \right)\mu_{l}]},\frac{-C_{1}+\alpha C_{1}+P_{c}[\left(1-\delta \right)\mu_{h}-\left(1+\delta \right)\mu_{l}]}{\delta G_{1}-C_{1}+\alpha C_{1}+P_{c}[\left(1-\delta \right)\mu_{h}-\mu_{l}]})$. When *N* < *N*_2_, TS becomes an evolutionarily stable strategy.

**Proposition 2.** When *δG*_1_ − *C*_1_ + *αC*_1_ + *P*_*c*_[*μ*_*h*_ − (1 + *δ*)*μ*_*l*_] < 0, *δG*_1_ − *C*_1_ + *αC*_1_ + *P*_*c*_[(1 − *δ*)*μ*_*h*_ − *μ*_*l*_] < 0, we have *N*_3_ that $N_{3}=\text{min}\;(\frac{-C_{1}+\alpha C_{1}+P_{c}[\left(1+\delta \right)\mu_{h}-\left(1+\delta \right)\mu_{l}]}{\delta G_{1}-C_{1}+\alpha C_{1}+P_{c}[\mu_{h}-\left(1+\delta \right)\mu_{l}]},\frac{-C_{1}+\alpha C_{1}+P_{c}[\left(1-\delta \right)\mu_{h}-\left(1+\delta \right)\mu_{l}]}{\delta G_{1}-C_{1}+\alpha C_{1}+P_{c}[\left(1-\delta \right)\mu_{h}-\mu_{l}]})$. When *N* < *N*_3_, LS becomes an evolutionarily stable strategy. There is *N*_4_ that $N_{4}=\text{max}(\frac{-C_{1}+\alpha C_{1}+P_{c}[\left(1+\delta \right)\mu_{h}-\left(1+\delta \right)\mu_{l}]}{\delta G_{1}-C_{1}+\alpha C_{1}+P_{c}[\mu_{h}-\left(1+\delta \right)\mu_{l}]},\frac{-C_{1}+\alpha C_{1}+P_{c}[\left(1-\delta \right)\mu_{h}-\left(1+\delta \right)\mu_{l}]}{\delta G_{1}-C_{1}+\alpha C_{1}+P_{c}[\left(1-\delta \right)\mu_{h}-\mu_{l}]})$. When *N* > *N*_4_, TS becomes an evolutionarily stable strategy.

Under strong selection, whether the service provider chooses LS is related to the group size, basic benefit of service providers providing low-carbon manufacturing services, low-carbon costs, cost-sharing coefficient, actual carbon emissions, carbon trading price of unit carbon emission, and matching growth rate. When the payoff of providers choosing LS is higher than that of choosing TS, the selection behavior supports LS to invade TS, and LS becomes an evolutionarily stable strategy. When their number of service providers is above *N*_1_ or below *N*_3_, service providers choose to provide low-carbon manufacturing services to maximize benefits.

### 4.2 Weak selection

Under weak selection, stochastic factors including social responsibility have a dominating effect on the low-carbon decision-making of service providers. The strategy selection depends almost entirely on stochastic factors, and the expected payoff has little effect on low-carbon decision-making [39]. Taylor series expansion is applied to Eqs (16) and (17).

$$\begin{array}{c} p_{L}=\frac{1}{1+\sum_{j=1}^{N-1}\;e^{w\sum_{k=1}^{j}(\pi_{k}^{T}-\pi_{k}^{L})}}=\frac{1}{N}+\frac{w}{6N}\left(a+bN\right) \end{array}$$ (23)

$$\begin{array}{c} p_{T}=\frac{1}{1+\sum_{j=1}^{N-1}\;e^{w\sum_{k=1}^{j}(\pi_{k}^{L}-\pi_{k}^{T})}}=\frac{1}{N}+\frac{w}{6N}\left(c+dN\right) \end{array}$$ (24)

Where *a* = 3*C*_1_ − 3*C*_1_*α* − 3*P*_*c*_*μ*_*h*_ + 3*P*_*c*_*μ*_*l*_ − *P*_*c*_*μ*_*h*_*δ* + *P*_*c*_*μ*_*l*_*δ*,

*b* = −3*C*_1_ + 3*C*_1_*α* + 3*P*_*c*_*μ*_*h*_ − 3*P*_*c*_*μ*_*l*_ + 3*δG*_1_ − *P*_*c*_*μ*_*h*_*δ* − 2*P*_*c*_*μ*_*l*_*δ*,

*c* = −3*C*_1_ + 3*C*_1_*α* + 3*P*_*c*_*μ*_*h*_ − 3*P*_*c*_*μ*_*l*_ + *P*_*c*_*μ*_*h*_*δ* − *P*_*c*_*μ*_*l*_*δ*,

*d* = 3*C*_1_ − 3*C*_1_*α* − 3*P*_*c*_*μ*_*h*_ + 3*P*_*c*_*μ*_*l*_ − 3*δG*_1_ + *P*_*c*_*μ*_*h*_*δ* + 2*P*_*c*_*μ*_*l*_*δ*.

Taking neutral invasion probability $\frac{1}{N}$ as the benchmark, the low-carbon strategy evolution is analyzed. If $p_{L}>\frac{1}{N}$, the selection favors LS to invade TS. If $p_{T}>\frac{1}{N}$, the selection favors TS to invade LS. The following two propositions are obtained.

**Proposition 3.** When $N>\frac{-3C_{1}+3C_{1}\alpha +3P_{c}\mu_{h}-3P_{c}\mu_{l}+3\gamma G_{1}+P_{c}\mu_{h}\delta -P_{c}\mu_{l}\delta +\delta \gamma G_{1}}{-3C_{1}+3C_{1}\alpha +3P_{c}\mu_{h}-3P_{c}\mu_{l}+3\gamma G_{1}+3\delta G_{1}-P_{c}\mu_{h}\delta -2P_{c}\mu_{l}\delta +2\delta \gamma G_{1}}$ (*b* > 0) or $N<\frac{-3C_{1}+3C_{1}\alpha +3P_{c}\mu_{h}-3P_{c}\mu_{l}+3\gamma G_{1}+P_{c}\mu_{h}\delta -P_{c}\mu_{l}\delta +\delta \gamma G_{1}}{-3C_{1}+3C_{1}\alpha +3P_{c}\mu_{h}-3P_{c}\mu_{l}+3\gamma G_{1}+3\delta G_{1}-P_{c}\mu_{h}\delta -2P_{c}\mu_{l}\delta +2\delta \gamma G_{1}}\left(b<0\right)$, $p_{L}>\frac{1}{N}$.

Proposition 3 indicates that natural selection supports LS to invade TS when the group size meets the above conditions, i.e., it favors service providers choosing LS to take root in the group so that low-carbon cooperation can succeed.

**Proposition 4.** When $N>\frac{3C_{1}-3C_{1}\alpha -3P_{c}\mu_{h}+3P_{c}\mu_{l}-3\gamma G_{1}-P_{c}\mu_{h}\delta -\delta \gamma G_{1}+P_{c}\mu_{l}\delta }{3C_{1}-3C_{1}\alpha -3P_{c}\mu_{h}+3P_{c}\mu_{l}-3\delta G_{1}-3\gamma G_{1}+P_{c}\mu_{h}\delta -2\delta \gamma G_{1}+2P_{c}\mu_{l}\delta }$ (*d* > 0) or $N<\frac{3C_{1}-3C_{1}\alpha -3P_{c}\mu_{h}+3P_{c}\mu_{l}-3\gamma G_{1}-P_{c}\mu_{h}\delta -\delta \gamma G_{1}+P_{c}\mu_{l}\delta }{3C_{1}-3C_{1}\alpha -3P_{c}\mu_{h}+3P_{c}\mu_{l}-3\delta G_{1}-3\gamma G_{1}+P_{c}\mu_{h}\delta -2\delta \gamma G_{1}+2P_{c}\mu_{l}\delta }\left(d<0\right)$, $p_{T}>\frac{1}{N}$.

Proposition 4 indicates that natural selection supports TS to invade LS when the group size meets the above conditions, i.e., TS prevails, and low-carbon cooperation fails.

Under weak selection, whether a service provider adopts a low-carbon strategy is related to the basic benefit of service providers providing low-carbon manufacturing services, low-carbon costs, cost-sharing coefficient, actual carbon emissions, carbon trading price of unit carbon emission, and matching growth rate when the number of providers remains unchanged. When the fixed probability of LS is higher than that of neutral selection, LS is dominant.

## 5 Simulation analysis

Based on the Moran process analysis of providers’ low-carbon strategies, the impacts of cost-sharing coefficient, matching growth rate, low-carbon cost, and carbon trading price on the strategy evolution are analyzed under strong selection and weak selection. In addition, the impacts of selection intensity and group size on the low-carbon strategy evolution of service providers are also analyzed.

According to the data in the literature [38, 40, 41], the following parameters are set: The basic benefit *G*_1_ of service providers providing manufacturing services is 20. The carbon quota *μ*_*p*_ for service providers is 10. When providers choose LS, their carbon emission *μ*_*l*_ is 8. The carbon emission *μ*_*h*_ of service providers choosing TS is 12. According to [38, 41], the carbon trading price *P*_*c*_ of unit carbon emission is 2. According to the above parameters and the fact that providers’ payment matrix in Table 3 is non-negative, the following parameters are assumed: The low-carbon cost *C*_*l*_ of service providers providing low-carbon manufacturing services is 10. The cost-sharing coefficient *α* is 0.2. Matching growth rate *δ* is 0.2. According to the definitions of Eqs (7) and (8), selection intensity *w* is 1 under strong selection, and selection intensity *w* is 0.0001 under weak selection.

### 5.1 Strategy evolution analysis of service providers under strong selection

**Fig 4:**
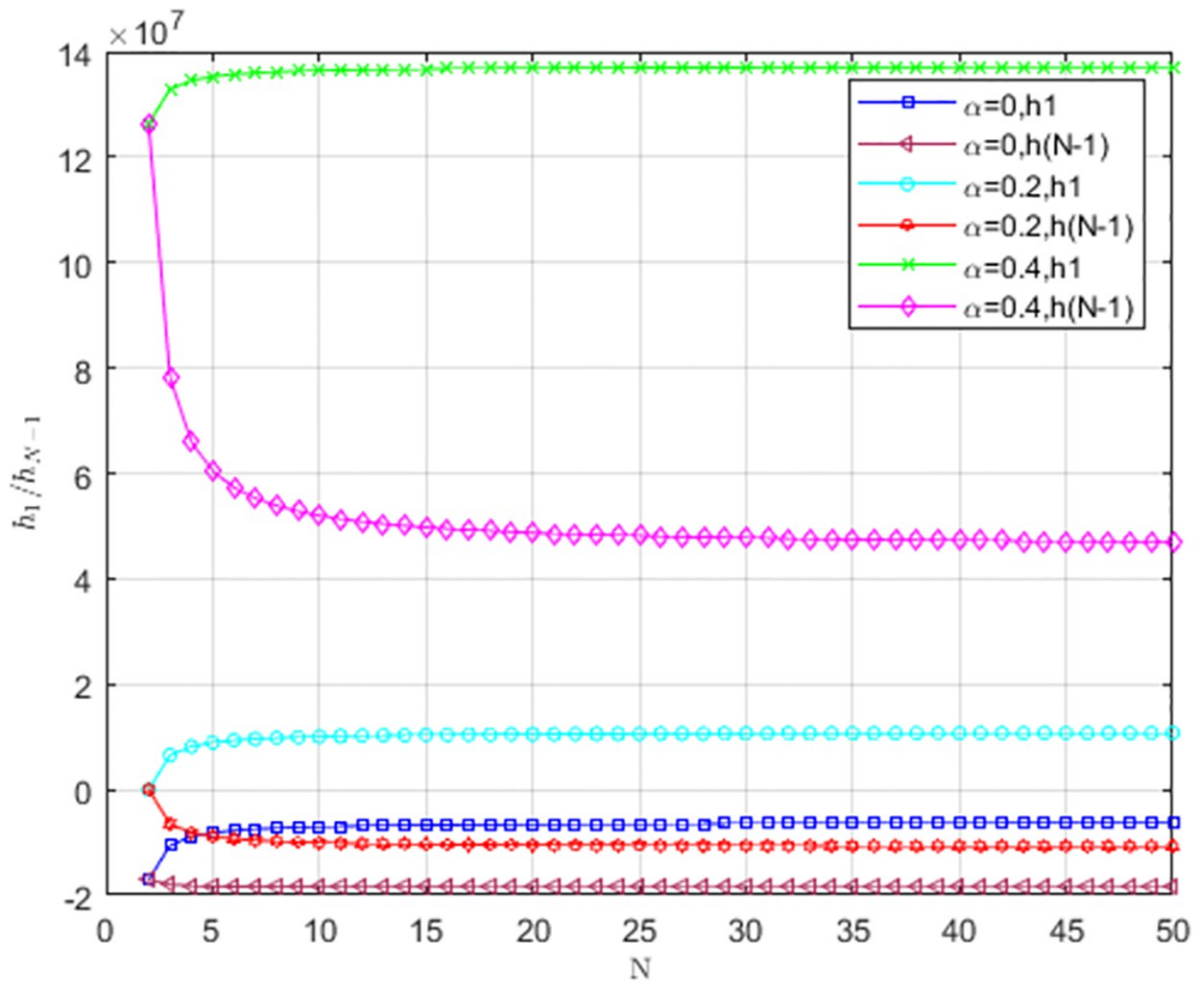
The impact of cost-sharing coefficient *α* on *h*_1_/*h*_*N*−1_.

The impact of cost-sharing coefficient *α* on *h*_1_/*h*_*N*−1_ is shown in Fig 4. When *α* = 0, *h*_1_ < 0, *h*_*N*−1_ < 0. The provider’s behavior is against LS to invade TS and supports TS to invade LS. Therefore, TS is dominant. When *α* = 0.2, *h*_1_ > 0, *h*_*N*−1_ < 0. The provider’s behavior supports LS to invade TS and supports TS to invade LS. The hybrid strategy is an evolutionarily stable strategy. When *α* = 0.4, *h*_1_ > 0, *h*_*N*−1_ > 0, LS prevails. As cost-sharing coefficient *α* decreases, two curves of *h*_1_, *h*_*N*−1_ are below *h*_1_ = 0, *h*_*N*−1_ = 0. TS replaces LS, and TS becomes an evolutionarily stable strategy. As the number of service providers increases, *h*_1_/*h*_*N*−1_ stabilizes to a fixed value.

As shown in Fig 4, service providers change from TS to LS as the cost-sharing coefficient increases. Therefore, it is necessary for the cloud platform to offer cost subsidies to service providers. With a higher cost-sharing coefficient, service providers are more willing to choose low-carbon strategies.

**Fig 5:**
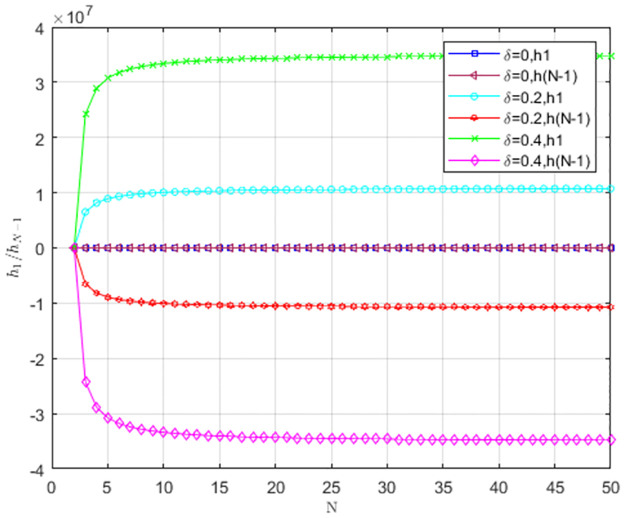
The impact of matching growth rate *δ* on *h*_1_/*h*_*N*−1_.

The impact of matching growth rate *δ* on *h*_1_/*h*_*N*−1_ is presented in Fig 5. As *δ* increases, *h*_1_ > 0, *h*_*N*−1_ < 0. Therefore, the stable strategy is a hybrid one, and the threshold that LS becomes an evolutionarily stable strategy is larger. According to Fig 5, matching growth rate does not change the evolutionarily stable strategy of providers.

As shown in Fig 5, the increase in matching growth rate does not change service providers’s strategy. Therefore, the matching growth rate does not promote the low-carbon behavior of service providers.

**Fig 6:**
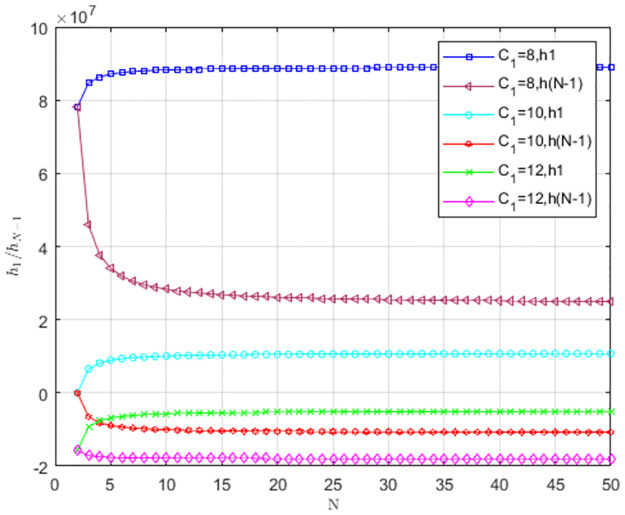
The impact of low-carbon cost *C*_1_ on *h*_1_/*h*_*N*−1_.

The impact of low-carbon cost *C*_1_ on *h*_1_/*h*_*N*−1_ is illustrated in Fig 6. When *C*_1_ = 12, *h*_1_ < 0, *h*_*N*−1_ < 0, and providers tend to choose TS. When *C*_1_ = 10, *h*_1_ > 0, *h*_*N*−1_ < 0, and the selection behavior supports the mutual invasion of LS and TS. When *C*_1_ = 8, *h*_1_ > 0, *h*_*N*−1_ > 0, and LS is more likely to become an evolutionarily stable strategy. As the low-carbon cost decreases, service providers change their strategy from TS to LS. With the increase of group size, the change of *h*_1_/*h*_*N*−1_ becomes smaller and gradually stabilizes to a fixed value.

As shown in Fig 6, with a lower low-carbon cost, service providers tend to choose low-carbon strategies.

**Fig 7:**
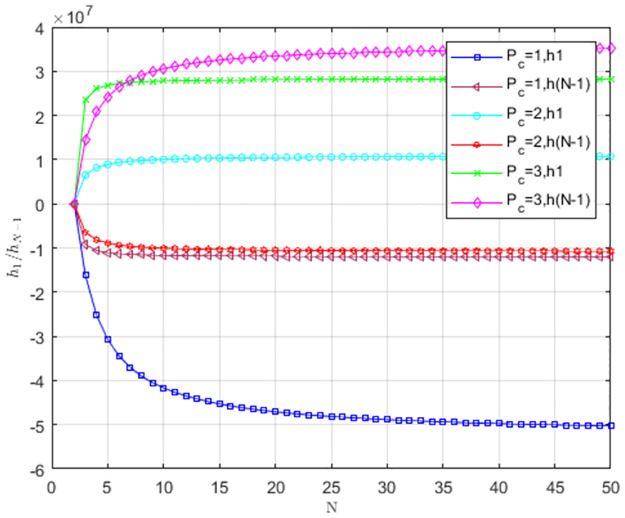
The impact of carbon trading price *P*_*c*_ on *h*_1_/*h*_*N*−1_.

Fig 7 reveals the impact of carbon trading price *P*_*c*_ on *h*_1_/*h*_*N*−1_. Carbon trading price affects the strategy selection of service providers. When *P*_*c*_ = 3, *h*_1_ > 0, *h*_*N*−1_ > 0, LS is dominant, and service providers tend to choose LS. With the decrease of *P*_*c*_, two curves of *h*_1_/*h*_*N*−1_ are below *h*_1_ = 0 and *h*_*N*−1_ = 0. TS is dominant, and TS is more likely to become an evolutionarily stable strategy.

Fig 7 shows that service providers are more inclined to choose low-carbon strategies as *P*_*c*_ increases.

### 5.2 Strategy evolution analysis of service providers under weak selection

**Fig 8:**
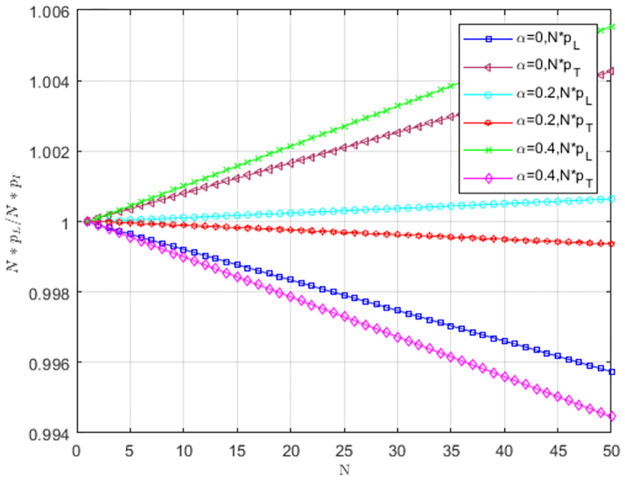
The impact of cost-sharing coefficient *α* on *NP*_*L*_/*NP*_*T*_.

The impact of cost-sharing coefficient *α* on *Np*_*L*_/*Np*_*T*_ is shown in Fig 8. When *α* = 0, $p_{L}<\frac{1}{N},p_{T}>\frac{1}{N}$. The selection behavior of the providers favors TS to replace LS, and TS is dominant. With the increase of *α*, $p_{L}>\frac{1}{N},p_{T}<\frac{1}{N}$. Service providers choosing LS occupy the entire group. When *α* = 0.2 and *α* = 0.4, LS is more likely to become an evolutionarily stable strategy as the number of providers increases.

**Fig 9:**
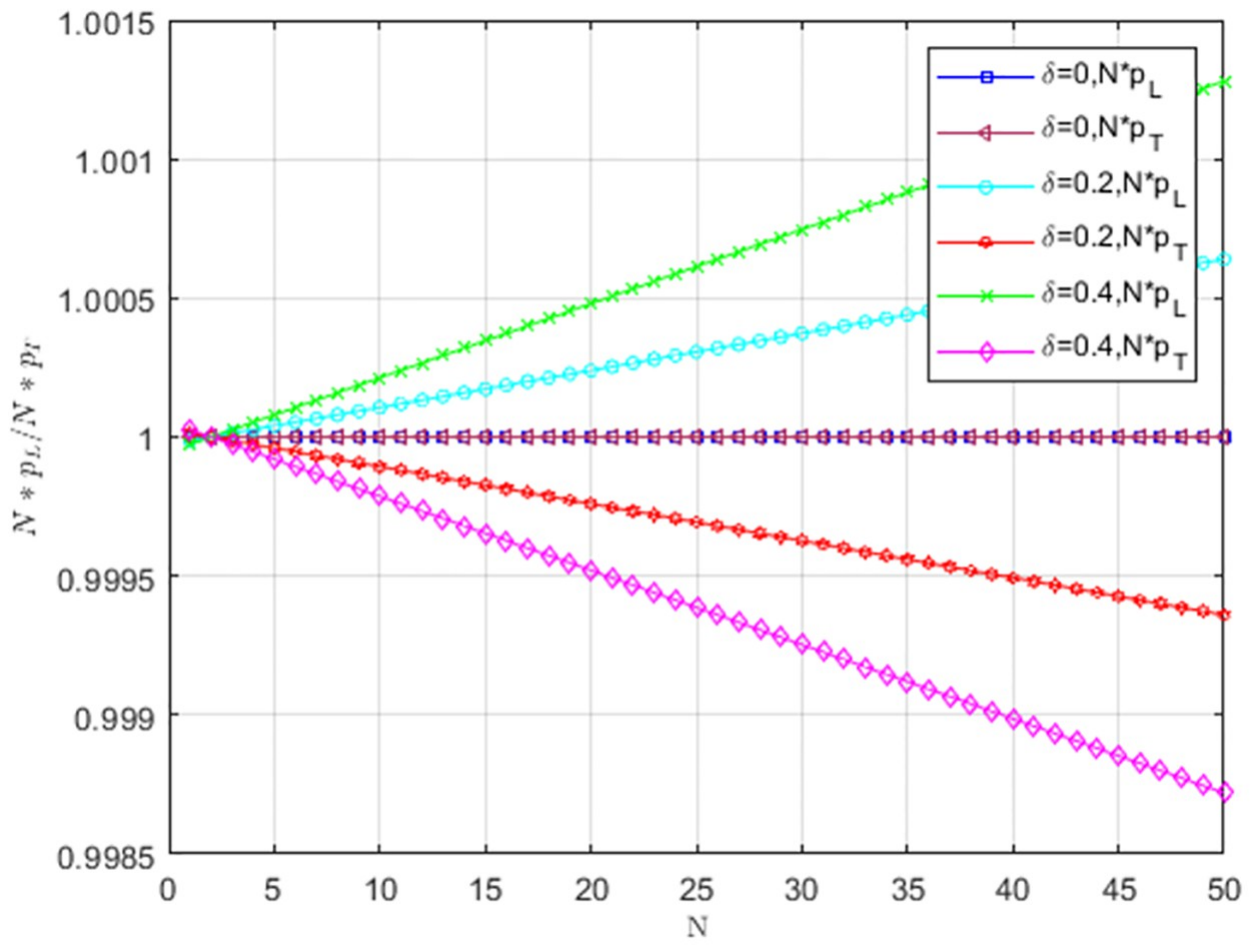
The impact of matching growth rate *δ* on *Np*_*L*_/*Np*_*T*_.

Fig 9 illustrates the impact of matching growth rate *δ* on *Np*_*L*_/*Np*_*T*_. When *δ* = 0.2 and *δ* = 0.4, $p_{L}>\frac{1}{N},p_{T}<\frac{1}{N}$. The selection behavior of service providers supports LS to replace TS, and LS prevails. With the increase of *δ*, service providers are more willing to provide low-carbon services, and providers choosing LS occupy the entire group. As the number of providers increases, LS is more likely to become an evolutionarily stable strategy.

**Fig 10:**
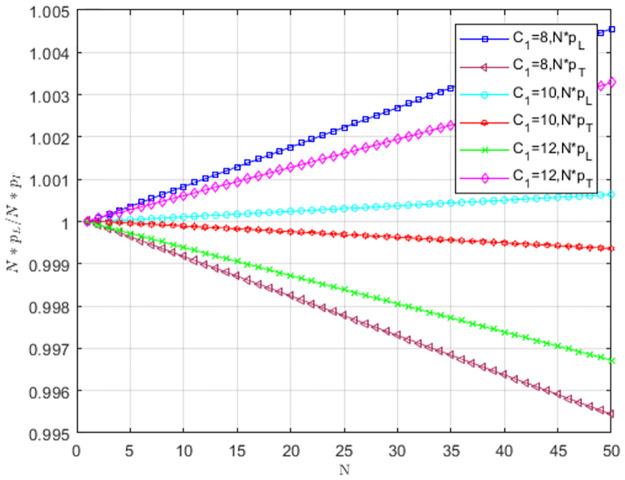
The impact of low-carbon costs *C*_1_ on *NP*_*L*_/*NP*_*T*_.

Under weak selection, the impact of low-carbon costs *C*_1_ on *Np*_*L*_/*Np*_*T*_ is shown in Fig 10. When *C*_1_ is larger, $p_{L}<\frac{1}{N},p_{T}>\frac{1}{N}$, the selection behavior of service providers supports TS to replace LS, and TS prevails. When low-carbon costs gradually decreases, $p_{L}>\frac{1}{N},p_{T}<\frac{1}{N}$. The selection behavior of service providers supports LS to replace TS. LS is dominant, and service providers tend to choose LS. When *C*_1_ = 8 and *C*_1_ = 10, LS is more likely to become an evolutionarily stable strategy as group size increases. The low cost can motivate service providers to choose LS, and the low-carbon strategy spreads when the size is larger.

**Fig 11:**
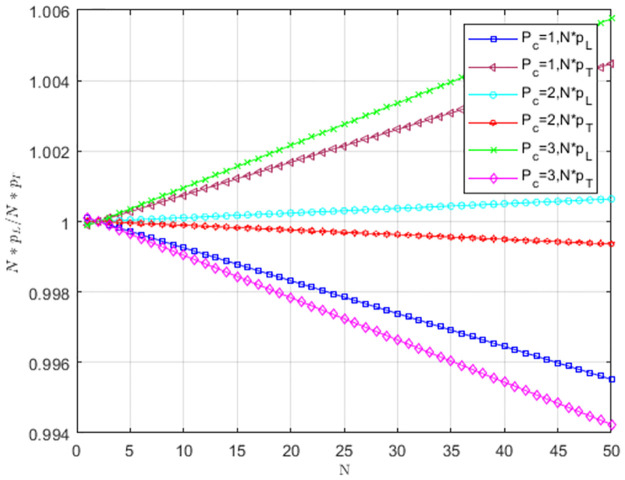
The impact of carbon trading price *P*_*c*_ on *Np*_*L*_/*Np*_*T*_.

Under weak selection, the impact of carbon trading price *P*_*c*_ on *Np*_*L*_/*Np*_*T*_ is shown in Fig 11. When *P*_*c*_ = 1, $p_{L}<\frac{1}{N},p_{T}>\frac{1}{N}$. The providers’ selection behavior does not favor LS to replace TS, and TS is an evolutionarily stable strategy. When *P*_*c*_ = 2 and *P*_*c*_ = 3, $p_{L}>\frac{1}{N},p_{T}<\frac{1}{N}$. The providers’ selection behavior favors LS to replace TS, and providers tend to choose LS. As *P*_*c*_ increases, service providers tend to choose LS. When *P*_*c*_ = 2 and *P*_*c*_ = 3, the low-carbon strategy is more likely to become a stable strategy as the group size increases.

### 5.3 The impacts of selection intensity and group size on the low-carbon strategy evolution of service providers

**Fig 12:**
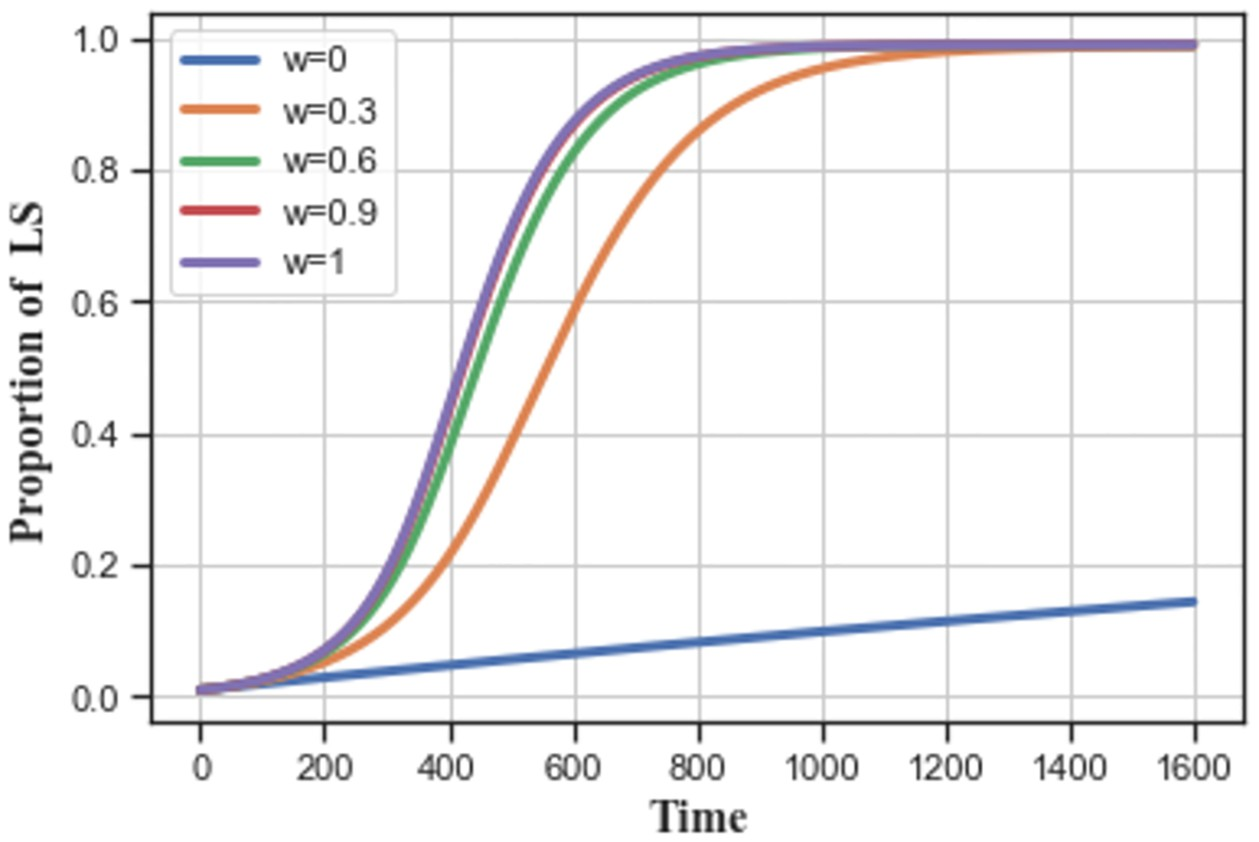
The impact of selection intensity *w* on low-carbon strategy evolution.

As *w* increases, service providers stabilize to equilibrium state ‘1’, which becomes shorter, as shown in Fig 12. Service providers are more likely to choose the low-carbon strategy. Therefore, benefit dominance can promote the diffusion of low-carbon strategies more than stochastic factor dominance in the group.

**Fig 13:**
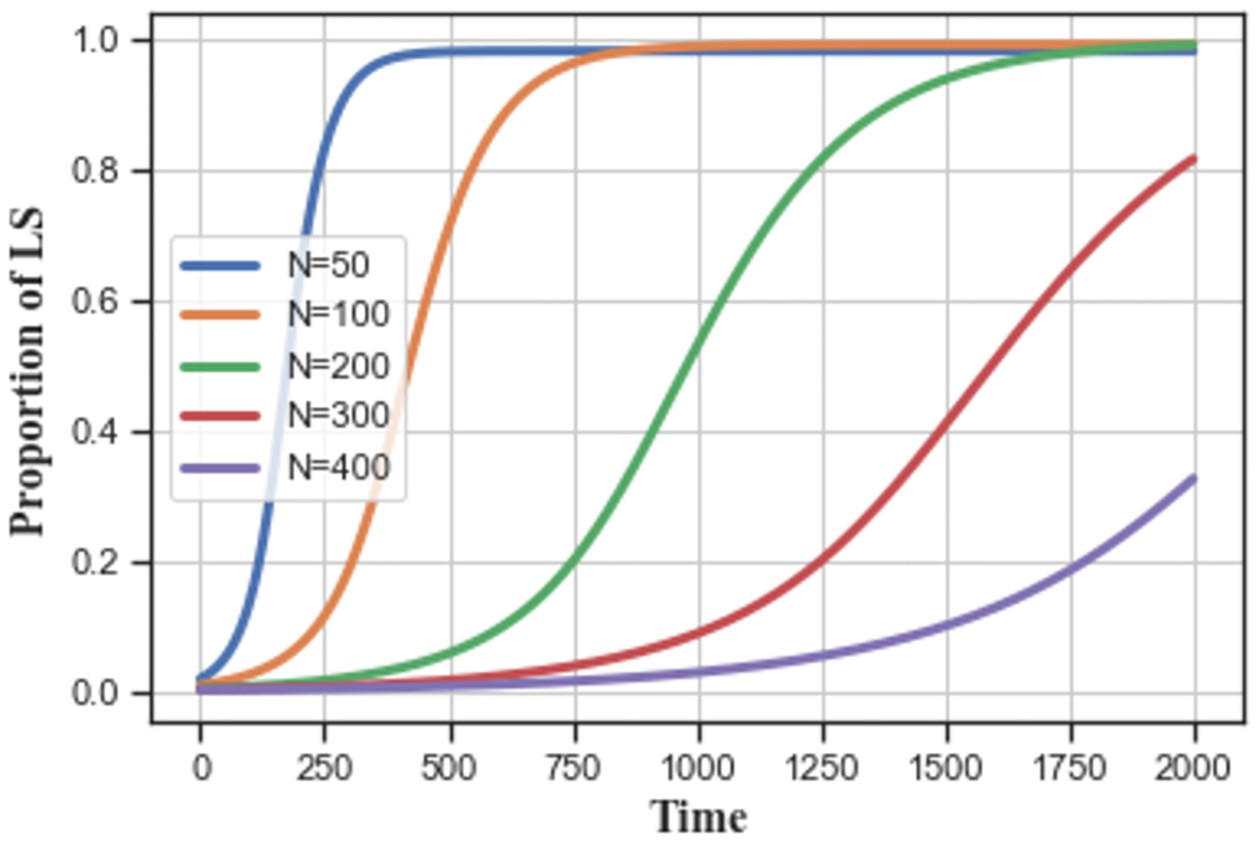
The impact of group size *N* on low-carbon strategy evolution.

As shown in Fig 13, service providers tend to choose low-carbon strategies as their number *N* decreases, and their evolution time to stabilize to equilibrium ‘1’ becomes shorter. With a larger group size, the low-carbon strategy spreads slower in the service provider group.

### 5.4 The combined effects of different factors on low-carbon strategy evolution of service providers

To visualize the combined effects of cost-sharing coefficient *α*, low-carbon cost *C*_1_, and carbon trading price *P*_*c*_, simulations are conducted with *α* changing from 0 to 1, *P*_*c*_ changing from 0 to 5, and *C*_1_ changing from 5 to 15.

**Fig 14:**
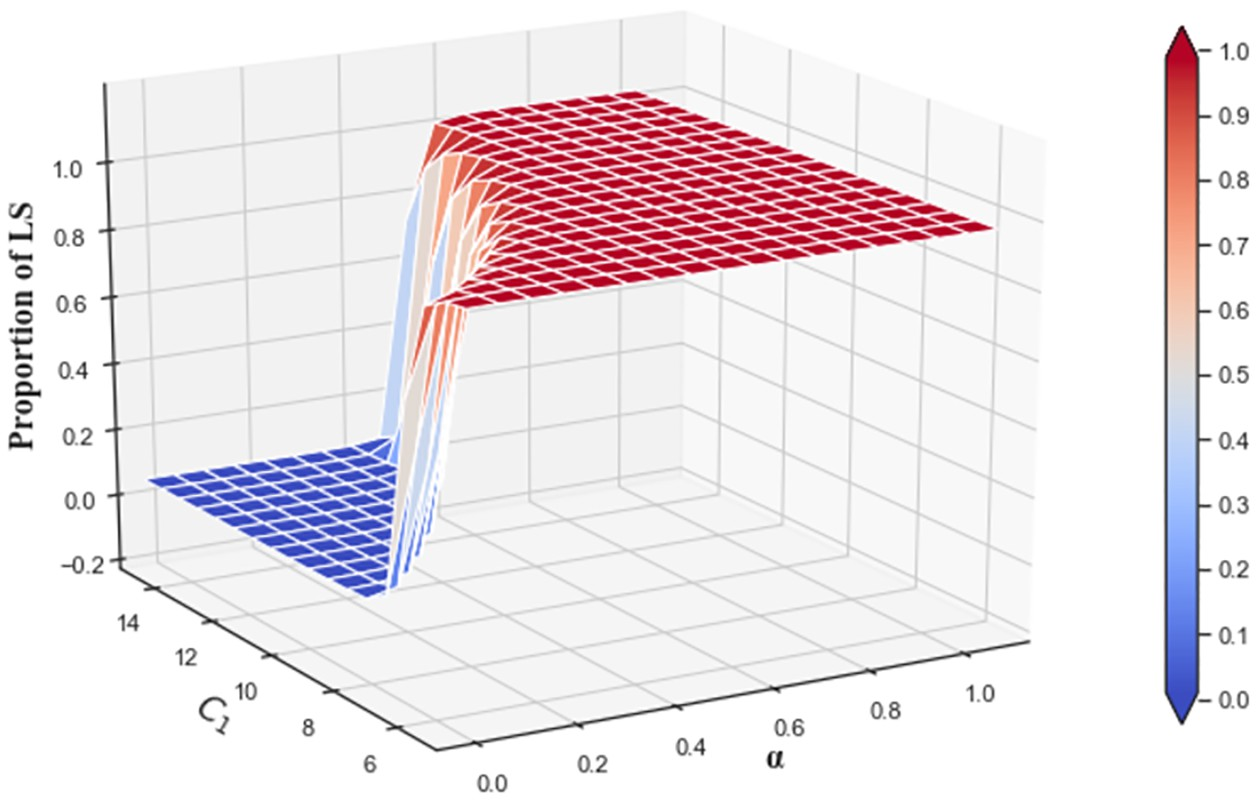
The combined effects of cost-sharing coefficient and low-carbon cost on low-carbon strategy evolution at *t* = 1000.

The combined effects of cost-sharing coefficient and low-carbon cost on the low-carbon strategy evolution are analyzed in Fig 14. A small cost-sharing coefficient *α* has an insignificant impact on the proportion of providers choosing LS when *C*_1_ is high. When *C*_1_ is below a certain threshold, the proportion rises faster as *α* increases, finally reaching about 0.99 at *t* = 1000.

The impact of the cost-sharing coefficient has a diminishing marginal effect, rendering the impact negligible with the cost-sharing coefficient continuously increasing at high values. In addition, the decrease in low-carbon cost causes the proportion rise to become gradual when *α* is larger. These results suggest that the combination of cost-sharing coefficient and low-carbon cost can have good effects on the evolution. However, the cloud platform still needs to consider the diminishing marginal effect of the cost-sharing coefficient when *C*_1_ is small.

**Fig 15:**
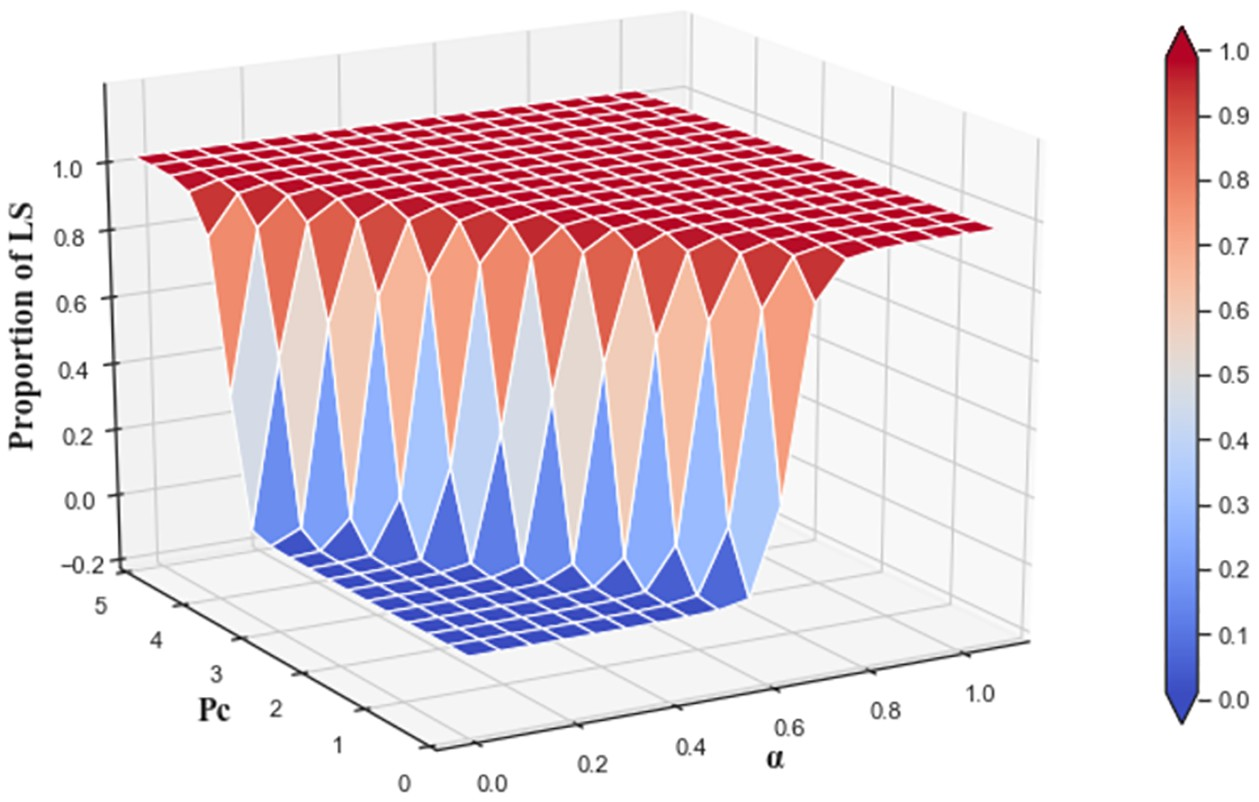
The combined effects of cost-sharing coefficient and carbon trading price on low-carbon strategy evolution at *t* = 1000.

The combined impact of the cost-sharing coefficient and carbon trading price on low-carbon strategy evolution is analyzed in Fig 15. When the cost-sharing coefficient *α* and carbon trading price *P*_*c*_ are small, the proportion of providers choosing LS is low. As *α* and *P*_*c*_ increase, the proportion reaches about 0.99 at *t* = 1000. The effects of cost-sharing coefficient and low-carbon cost have diminishing marginal effects. When *P*_*c*_ is small, the proportion of providers choosing LS can rise from 0 to 0.99 as *α* increases from 0 to 1. As *P*_*c*_ increases, the effect of *α* has a diminishing marginal effect.

**Fig 16:**
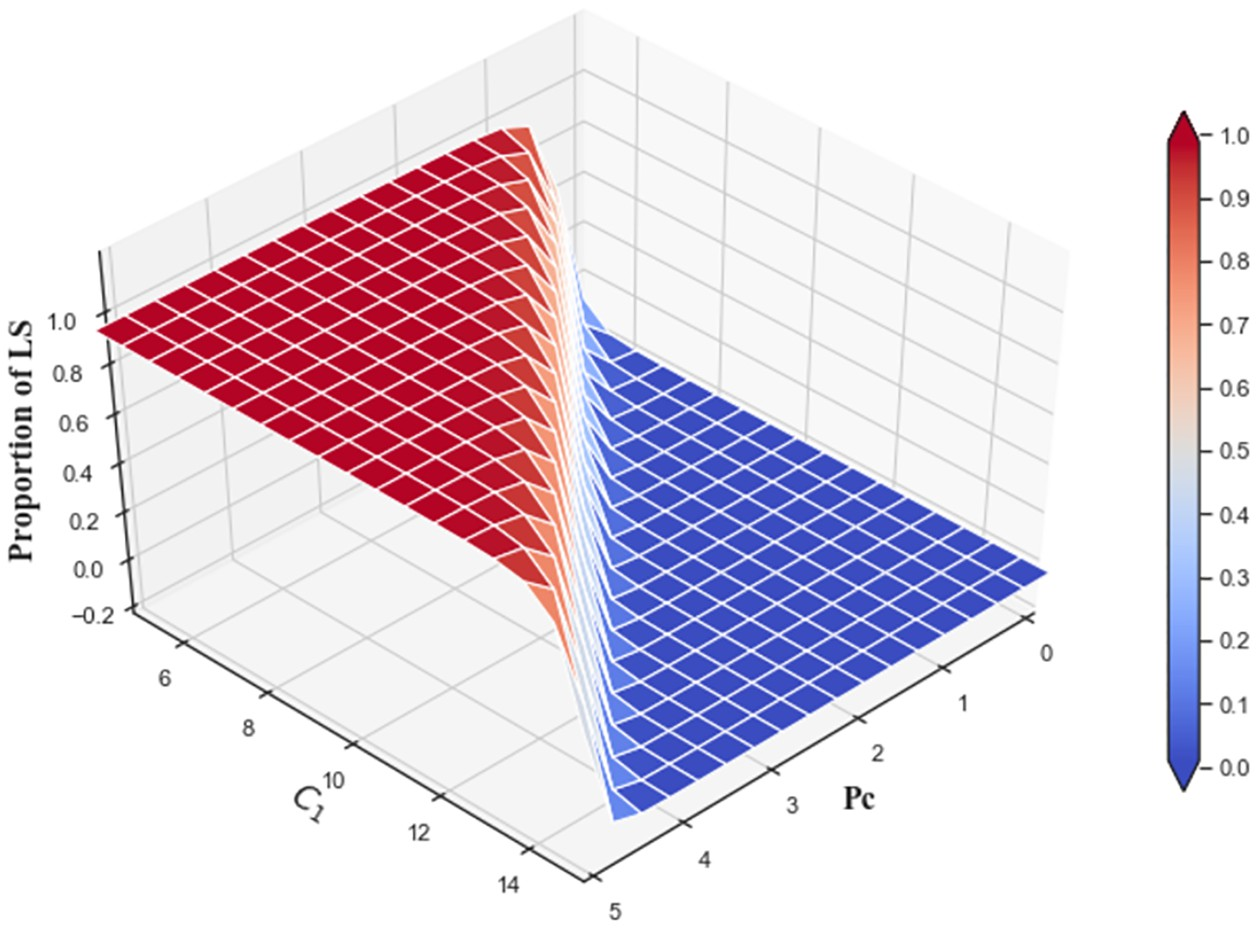
The combined effects of low-carbon cost and carbon trading price on low-carbon strategy evolution at *t* = 1000.

The combined effects of low-carbon cost and carbon trading price on low-carbon strategy evolution are shown in Fig 16. When *C*_1_ is small, the proportion rises from 0 to 0.99 with *P*_*c*_ increasing from 0 to 5. As *C*_1_ increases, the marginal effect of *P*_*c*_ is decreases. When *P*_*c*_ = 5 and *C*_1_ = 15, the proportion reaches about 0.1.

## 6 Discussions

The above simulation analysis explores the low-carbon strategy evolution process of service providers from four aspects. (1) Under strong selection, the expected payoff is dominant. The low-carbon strategy selection of service providers is related to their low-carbon cost, the cost-sharing coefficient of the cloud platform, and the carbon trading price of the government. When the group size of service providers is fixed, the service providers are more willing to provide low-carbon manufacturing services under greater low-carbon cost subsidies of the cloud platform, lower low-carbon costs, and higher carbon trading prices, that is, providers choosing low-carbon services occupy the entire group. (2) Under weak selection, stochastic factors are dominant. The main factors affecting the low-carbon strategy selection of service providers are low-carbon costs, cost-sharing coefficient, carbon trading price, and matching growth rate. When the group size of service providers is fixed, more providers tend to choose low-carbon strategies under greater cost subsidies of the cloud platform, higher matching growth rate of manufacturing services, lower low-carbon costs, and higher carbon trading prices. (3) Selection intensity is also an important factor affecting the strategy selection of service providers. As selection intensity increases, i.e., with a smaller impact of stochastic factors on their decision-making, service providers tend to choose low-carbon strategies. Moreover, with the increase in group size, the evolution time to reach stability is longer. (4) Compared to single factors, the combination of the cost-sharing coefficient, low-carbon cost, and carbon trading price can have better effects on the low-carbon strategy evolution of service providers.

Existing research mostly constructs static optimization models from the perspective of supply chain low-carbon coordination mechanisms and pricing/carbon emission reduction decisions. This study focuses on bounded rationality and stochastic factors in providers’ low-carbon decision-making process and conducts an evolutionary game analysis of providers’ low-carbon cooperation from a dynamic perspective. This study makes up for the research gap in the analysis of the low-carbon cooperation of cloud manufacturing service providers. Additionally, Moran process has been widely used in social stability management [31], such as epidemic prevention control [42], public opinion dissemination [43], and product quality decision-making [29]. This study applies the Moran process to low-carbon cooperation among providers, thus enriching its application scope.

## 7 Conclusions

Considering the stochastic factors in the low-carbon cooperation of service providers, this study analyzes the low-carbon strategy evolution process of providers based on the Moran process and discusses how important factors affect the low-carbon selection of service providers. The conclusions are as follows:

1. The factors influencing whether service providers choose a low-carbon strategy include low-carbon investment cost, cost-sharing coefficient of the cloud platform, carbon trading price, and matching growth rate of manufacturing services. As the cost-sharing coefficient of the cloud platform, carbon trading price, and matching growth rate increase, service providers are more inclined to choose low-carbon cooperation. Low-carbon investment costs have a negative impact on providers’ low-carbon cooperation.
2. Stochastic factors inhibit low-carbon cooperation among service providers. Additionally, as the group size increases, the time to stabilize into low-carbon cooperation increases.
3. The combination of cost-sharing coefficient, low-carbon investment cost, and carbon trading price can better motivate service providers to choose low-carbon strategies, but there is an attenuated marginal effect.

**Managerial implication**

1. The government should strengthen the publicity of environmental protection and improve environmental awareness of service providers. Additionally, the government should appropriately raise carbon trading prices in the carbon trading market to encourage low-carbon cooperation of service providers.
2. The cloud platform can bear part of the low-carbon costs of service providers. When choosing to provide low-carbon investment services, service providers need to bear additional costs, such as new equipment costs. Low-carbon investment is a long process, and the benefits can not be obtained in the short term. Even with a large initial proportion of service providers choosing low-carbon strategies, it is difficult to stabilize into low-carbon strategies when the cost-sharing of the cloud platform is small. Therefore, the cloud platform should offer appropriate cost subsidies to encourage service providers in low-carbon cooperation.
3. The joint incentives between the government and the cloud platform can better encourage service providers to participate in low-carbon cooperation, but the combination has a diminishing marginal effect. Therefore, it is necessary to set reasonable carbon trading prices and cost subsidies according to different conditions.

**Practical/Social implications**

The research results can provide suggestions for the government and the cloud platform to encourage service providers’ participation in low-carbon cooperation within uncertain environments, provide a basis for providers to form long-term win-win low-carbon cooperation relationships, and effectively promote the low-carbon and sustainable development of the cloud manufacturing supply chain, which has practical significance for environmental improvement.

This study still has some limitations. (1) This research analyzes low-carbon cooperation among service providers under government regulation and cloud platform’ incentives. In the future, low-carbon behavior can be analyzed from the perspective of service demanders. (2) Some parameters are set according to relevant literature during the simulation. Future research can validate the stochastic evolution model based on actual service provider data. (3) The correlation between digital technology and low-carbon cooperation in cloud manufacturing and how providers’ low-carbon decisions evolve under digital transformation can be explored.

## Supporting information

S1 Data (ZIP)
